# Selective Analysis of Sulfur-Containing Species in a Heavy Crude Oil by Deuterium Labeling Reactions and Ultrahigh Resolution Mass Spectrometry

**DOI:** 10.3390/ijms161226205

**Published:** 2015-12-17

**Authors:** Xuxiao Wang, Wolfgang Schrader

**Affiliations:** Max-Planck-Institut für Kohlenforschung, Kaiser-Wilhelm-Platz 1, D-45470 Mülheim an der Ruhr, Germany; wxuxiao@mpi-muelheim.mpg.de

**Keywords:** selective analysis, sulfur, crude oil, deuterium labeling, ultrahigh resolution, mass spectrometry

## Abstract

A heavy crude oil has been treated with deuterated alkylating reagents (CD_3_I and C_2_D_5_I) and directly analyzed without any prior fractionation and chromatographic separation by high-field Orbitrap Fourier Transform Mass Spectrometry (FTMS) and Fourier Transform Ion Cyclotron Resonance Mass Spectrometry (FT-ICR MS) using electrospray ionization (ESI). The reaction of a polycyclic aromatic sulfur heterocycles (PASHs) dibenzothiophene (DBT), in the presence of silver tetrafluoroborate (AgBF_4_) with ethyl iodide (C_2_H_5_I) in anhydrous dichloroethane (DCE) was optimized as a sample reaction to study heavy crude oil mixtures, and the reaction yield was monitored and determined by proton nuclear magnetic resonance spectroscopy (^1^H-NMR). The obtained conditions were then applied to a mixture of standard aromatic CH-, N-, O- and S-containing compounds and then a heavy crude oil, and only sulfur-containing compounds were selectively alkylated. The deuterium labeled alkylating reagents, iodomethane-d_3_ (CD_3_I) and iodoethane-d_5_ (C_2_D_5_I), were employed to the alkylation of heavy crude oil to selectively differentiate the tagged sulfur species from the original crude oil.

## 1. Introduction

The demand for affordable and reliable energy leads to a continuous focus on fossil-based materials, and nowadays the trend is shifting to heavier petroleum resources. One of the huge disadvantage of heavy crude oils as energy supply is that they contain rich heteroatoms, such as sulfur, nitrogen and oxygen. The sulfur content is detrimental to refining processes and harmful to the environment after combustion, and thus must be removed. Various stringent legislations and regulations have been implemented to limit the sulfur content of fuels [1]. A better understanding of heavy crude oil composition is necessary for that aim.

Heavy petroleum is a supercomplex [1] mixture of hydrocarbons containing various amounts of heteroatoms (N, O and S), and it challenges and meanwhile promotes the development of analytical techniques [2]. Mass spectrometry has been established as the most powerful and promising method to characterize such complex mixtures [3,4]. FT-ICR MS provides sufficient mass resolving power and mass accuracy to identify each of the thousands of different molecules and their elemental compositions from the most complex mixtures [5]. Recently, a new commercially available type of the high-field Orbitrap FTMS, the Orbitrap Elite [6,7,8], has been evaluated and employed successfully to analyze the petroleum samples with a resolving power of up to 900,000 at *m*/*z* 400 [9,10].

Electrospray ionization (ESI) [11] coupled with ultrahigh resolution mass spectrometry [9,12,13] is an excellent method employed to ionize polar species in crude oil. It efficiently ionizes the acidic or basic molecular species in petroleum by deprotonation or protonation to form [M − H]^−^ or [M + H]^+^ ions [11,12,14] and even highly condensed polyaromatic compounds can be ionized by ESI as radical cations [15].

However, PASHs are not basic enough to be efficiently ionized by ESI. Thus, some achievements have been made to enhance ionizing efficiency of nonpolar sulfur species in ESI process. Muller *et al.* [16] developed a derivatization method by forming the polar sulfonium salts in solution prior to ESI, but the selectivity toward sulfur aromatics is achieved by relying on chromatographic separations. Purcell *et al.* [17] derivatized a vacuum bottom bitumen residue by this derivatization procedure, and observed not only sulfur compounds but also detected nitrogen and other heteroatom containing classes at high abundance by positive ESI FT-ICR MS.

Metal complexation methods, such as Pd^2+^ electrospray mass spectrometry (ESI-MS) [18] and especially Ag^+^ ESI-MS [19], have also been developed for the detection of sulfur-containing compounds in petroleum by the formation of positive metal-complexes [20]. However, the relatively flexible coordination sphere of the metal ions allows them to coordinate with a variety of ligands even the solvents with the coordination numbers from 2 to 6, which on some level, increases the complexity of crude oil analysis [19,21,22].

Here, on the basis of previous research, we combine the silver coordination chemistry and nucleophilic chemistry of heteroatoms compounds with ESI-MS, and introduce the deuterium labeling technique to develop a method that allows determination of sulfur-containing species in a whole crude oil with high selectivity, and avoiding the complexity from the transition metal complexation. When using the derivatization procedure with methyl iodide in a crude oil sample, where the elements C, H, O, N, S and a few metals are usually present, it is difficult to separate the derivatized from the non-derivatized compounds. The difference cannot really be established because the derivatizing group contains the same elements as the compounds present in a crude oil sample and only the increase in signal intensity of sulfur compounds can be an indication of the reaction. Here, the usage of a deuterated reactant for the derivatization is introduced which allows to clearly distinguish the reacted from potentially unreacted compounds and allows an unambiguous characterization.

## 2. Results and Discussion

Ultrahigh-resolution MS has an unparalleled advantage for crude oil analysis, but as of yet, not all compositions present in heavier petroleum can be completely and accurately analyzed by any single available analytical method. Thus, simplification methods [23,24] and methods for selective analysis need to be developed to gain a deeper insight into crude oil composition. It has been shown that Ag^+^ ions kinetically coordinate preferentially to sulfur atoms instead of oxygen or nitrogen atoms to form metal-ligand bonds [20,25]. This Ag^+^ selectivity towards sulfur species can now be used for selective reactions with derivatization agents to tag sulfur heterocycles in crude oil. To achieve this goal, the reaction has been investigated in detail in three steps: (1) The reaction was carried out using a standard sulfur-containing compound. Here, dibenzothiophene (DBT) was chosen as a probe to optimize the reaction condition and monitored by proton nuclear magnetic resonance (^1^H-NMR) and ultrahigh resolution MS; (2) In the second stage the obtained conditions were applied for a simple mixture of standard aromatic CH-, N-, O- and S-containing compounds to investigate the selectivity; (3) Finally, the conditions were applied to a heavy crude oil.

It is worth noting that the feeding sequence plays an important role on the selectivity towards sulfur. Here, a certain amount of AgBF_4_ was added into the mixture of standards or crude oil, by which Ag^+^ is first allowed to selectively coordinate with sulfur atoms to form complex Ag^+^ adducts over a short time [26], then the alkylating reagent was added dropwise to the system and the S-C covalent bond was formed by the strong driving force of the precipitation of silver iodide (AgI). This derivatization procedure allows detection of sulfur compounds with very high selectivity, and meanwhile avoids the extra complications arising from silver complexation [22,27] and silver natural isotopes (51.84% ^107^Ag and 48.16% ^109^Ag) [20]. The implementation of multi-chemical methods combined with the ultrahigh resolution mass spectrometry (FT-ICR MS and Orbitrap FTMS) with electrospray ionization offers an efficient and feasible approach to detect the sulfur-containing species directly from the heavy petroleum without any time-consuming fractionation and separation.

### 2.1. Ethylation of Dibenzothiophene (DBT)

The standard derivatization reaction that was previously introduced [16] was carried out under the following condition: containing 10^−2^ and 4 × 10^−3^ mmol sulfur and 1 mmol of CH_3_I, were dissolved in 3 mL of dry DCE. A solution of 1 mmol AgBF_4_ in 2 mL of DCE was then added. While the initial reaction that was reported by Muller *et al.* [16] was only implemented for the derivatization with CH_3_I; here an additional derivatization agent, C_2_H_5_I, was studied as ethylating agent. In addition, the reaction was carried out using both reagents in the deuterated form to directly study the reaction product and distinguish derivatized from non-derivatized compounds in the crude oil sample that was not chromatographically simplified.

**Figure 1:**
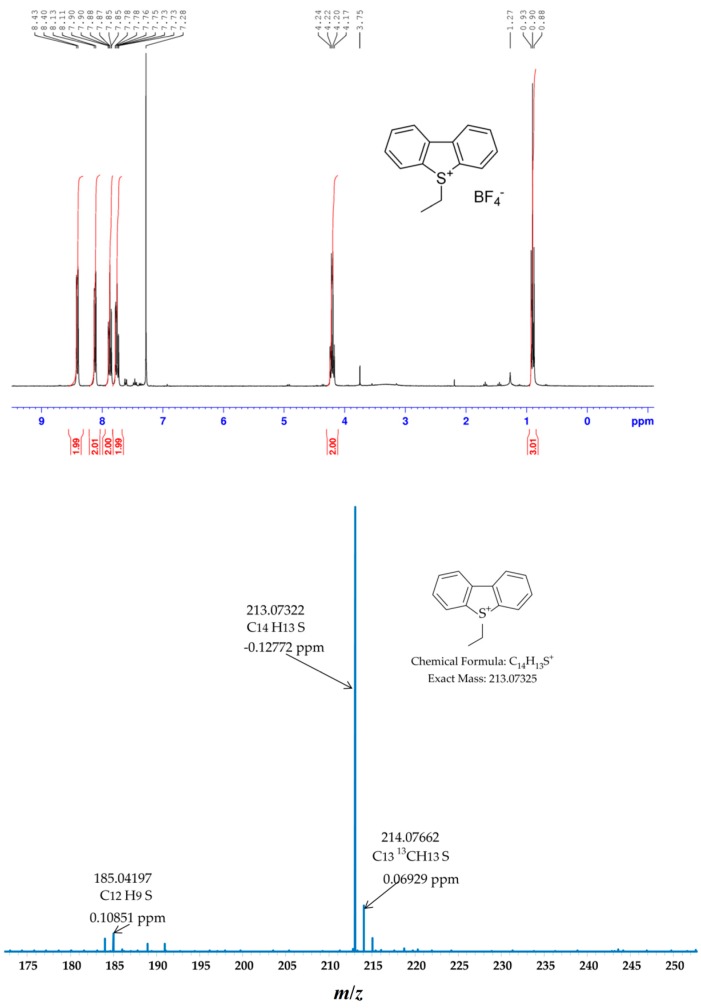
^1^H-NMR spectra (**top**) and HRMS (electrospray ionization Fourier transform ion cyclotron resonance mass spectrometry (ESI FT-ICR MS)) spectra (**bottom**) of 5-ethyldibenzo[*b*,*d*]thiophenium tetrafluoroborate.

**Scheme 1:**
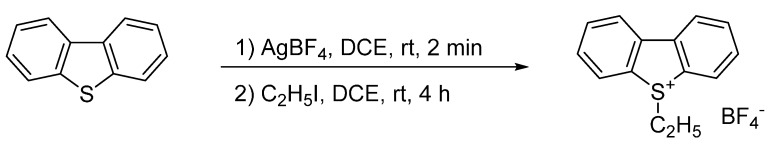
Ethylation of dibenzothiphene (DBT), reaction carried out at room temperature (rt).

The molar ratio of DBT, CH_3_I (or C_2_H_5_I) and AgBF_4_ remains 1:2:2, and the yield of crude product determined by ^1^H-NMR was 70% for CH_3_I and 87% for C_2_H_5_I. Similar experimental results were obtained by Acheson *et al.* [28], so C_2_H_5_I was chosen for the continuous studies. In regard to selectivity, the reaction needed to be altered. Instead of adding equal molar ratio of CH_3_I and AgBF_4_ (1 mmol) that are 250 times the concentration of sulfur, as mentioned by Muller *et al.*, [16] the reaction condition depicted in Scheme 1 were optimized to produce a high yield of sulfonium salt.

When the molar ratio of DBT, AgBF_4_ and C_2_H_5_I remains 1:2:8, *i.e.*, the amount of AgBF_4_ is only 2 times more than DBT, the yield determined by ^1^H-NMR increases significantly (99%), as shown in Figure 1 (top).

The ^1^H-NMR spectrum of the above salt is simple because of the molecular symmetry (see Figure 1 top). The 4 groups of protons with the integral ratio 2:2:2:2 in the downfield belong to the aromatic rings; the 2 groups of protons with the integral ratio 2:3 in the upfield are attributed to the ethyl group. With the appropriate splitting according to the relevant environments of each proton, the structure of 5-ethyldibenzo[*b*,*d*]thiophenium tetrafluoroborate was ambiguously determined. ^1^H-NMR (300 MHz, CDCl_3_): δ 8.41 (d (doublet), *J* = 7.6 Hz, 2H, 2 × *Ph*), 8.12 (d, *J* = 7.1 Hz, 2H, 2 × *Ph*), 7.87 (t (triplet), *J* = 7.6 Hz, 2H, 2 × *Ph*), 7.76 (t, *J* = 7.9 Hz, 2H, 2 × *Ph*), 4.21 (q (quartet), *J* = 7.1 Hz, 2H, 2 × CH*_2_*CH_3_), 0.90 (t, *J* = 7.1 Hz, 3H, 3 × CH*_2_*CH_3_).

The ethylated DBT salt, 5-ethyldibenzo[*b*,*d*]thiophenium tetrafluoroborate was obtained as colorless needles. It was determined by the positive HRMS (ESI FT-ICR MS) in Figure 1 (bottom) at *m*/*z* 213.07322 (calcd 213.07325) for C_14_H_13_S^+^. A tiny peak of [M + H] was also observed at *m/z* 185.04197, and explanation might be given by the loss of the ethyl group under ESI condition. It was shown, that when using a heated nebulizer the derivatization group can be removed during the ionization process [1].

### 2.2. Ethylation of a Mixture of Standards (ANTH, DBT, ACR and DBF)

**Figure 2:**
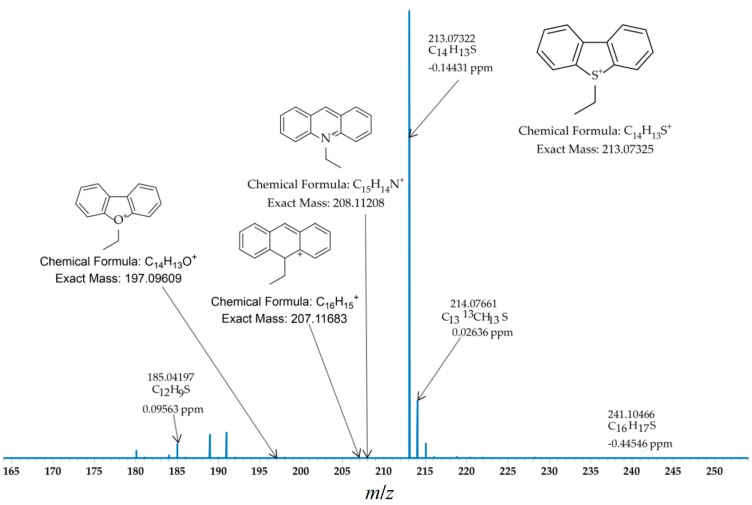
ESI FT-ICR MS spectrum of a mixture of the ethylated standards (anthracene (ANTH), DBT, acridine (ACR) and dibenzofuran (DBF)).

Based on the results of the reaction of a pure standard sulfur compound, the conditions were applied to a mixture of standards (antracene (ANTH), dibenzothiphene (DBT), acrinine (ACR) and dibenzofuran (DBF)). The HR ESI-MS spectrum of the ethylated mixture of standards was obtained as shown in Figure 2. No corresponding signals of derivatized ANTH, ACR and DBF were observed (calcd. *m*/*z* values at 207.11683, 208.11208 and 197.09609), and only DBT was selectively ethylated among this standards mixture. Besides the peaks from DBT, a very minor signal [M + H]^+^ from underivatized ACR at *m*/*z* 180.08084 was detected. Additionally, minor traces of the disubstituted product of DBT was observed as well. From the results obtained above, we can see, even in the presence of other polyromantic heterocyclic compounds, the nucleophilic substitution of sulfur aromatic compound works with high selectivity towards sulfur species due to activation by the silver ions.

### 2.3. Methylation and Ethylation of a Heavy Crude Oil

Deuterated alkylating reagents (CD_3_I and C_2_D_5_I) were utilized to alkylate the whole heavy crude oil instead of the standard reagents to distinguish the compounds present in a crude oil that contain CH-, N-, S- and O- elements from the derivatized ones. An adjustment for CD_3_I had to be made to a molar ratio of crude oil, AgBF_4_ and CD_3_I of 1:2:10.

**Figure 3:**
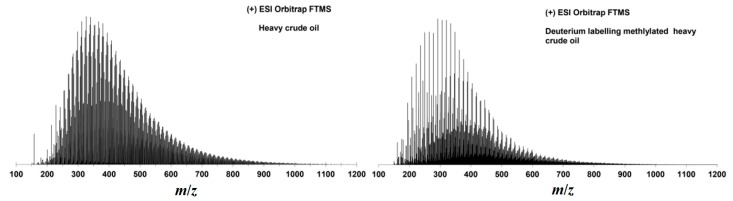
ESI(+) Orbitrap FTMS mass spectrum of heavy crude oil (**left**) and deuterium labeling methylated heavy crude oil (**right**).

**Figure 4:**
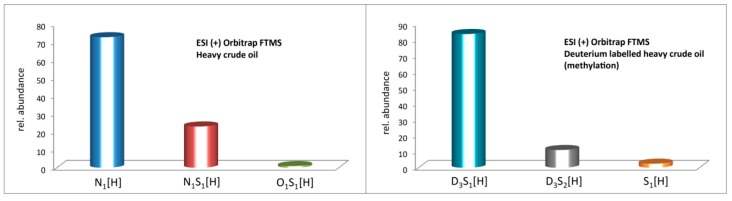
Class distribution for heavy crude oil from direct analysis (**left**) and heavy crude oil after deuterium labelled methylation (**right**).

Positive ESI Orbitrap FTMS analysis was performed for the untreated and methylated heavy crude oil. For a better signal-to-noise ratio, up to 500 scans in the mass range of *m*/*z* 150–1200 were accumulated, and a difference of the mass spectra before and after methylation can be seen in Figure 3. It is worth noting that the dramatic change of heteroatom class distribution before and after deuterium labeled methylation in Figure 4 is immense. Before the derivatization reaction, the more polar compounds are detected as expected. Here, the majority of signals that were assigned belong to the N_1_[H] class, with additional signals from the N_1_S_1_[H] and in very minor amounts from the O_1_S_1_[H] class. Note that the protonated molecules that were detected are distinguished by [H]. Radial ions would be described without [H].

After methylation, the compound classes that were assigned changed drastically. Now, instead of the polar compounds that are usually ionized by ESI, the nonopolar S_1_ and S_2_ classes which are usually not ionized by ESI are detected at high abundance. The deuterated version of the derivatization agent now also makes it possible to readily distinguish that the sulfur species detected are arising from the derivatization reaction and not only from the crude oil. After the reaction the sulfur species are present as *S*-methyl (*d_3_*) sulfonium salts. A small amount of S_1_ compounds (2.6%) can also be detected.

**Figure 5:**
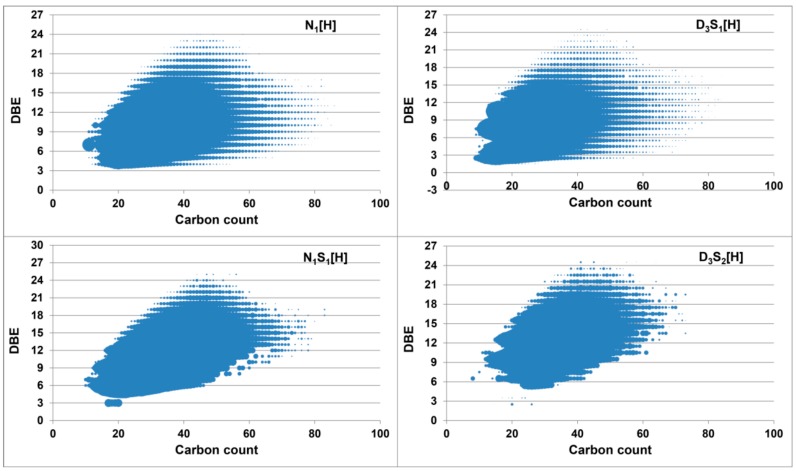
Double bond equivalent (DBE) *versus* carbon number plots for the N_1_[H] and N_1_S_1_[H] classes from the protonated heavy crude oil (**left**) and for the D_3_S_1_[H] and D_3_S_2_[H] classes from the deuterated methylated heavy crude oil (**right**). Note that the [H] indicates a protonated molecule.

The details of the results are shown in Figure 5. The N_1_[H] class in the non-derivatized sample generated from protonation has a DBE range between 4 and 24 with a carbon number distribution from 11 to 86. The N_1_S_1_[H] class meanwhile has a DBE range of 5–25 and carbon number of 10–83. The deuterated S_1_[H] class spans a range of DBE (2–25) and carbon number (9–84), where the compounds with DBE value of 2 are assumed to be the aliphatic sulfides containing a ring or a double bond. The deuterated S_2_[H] class has a DBE range of 3–25 and carbon number (8–73).

**Figure 6:**
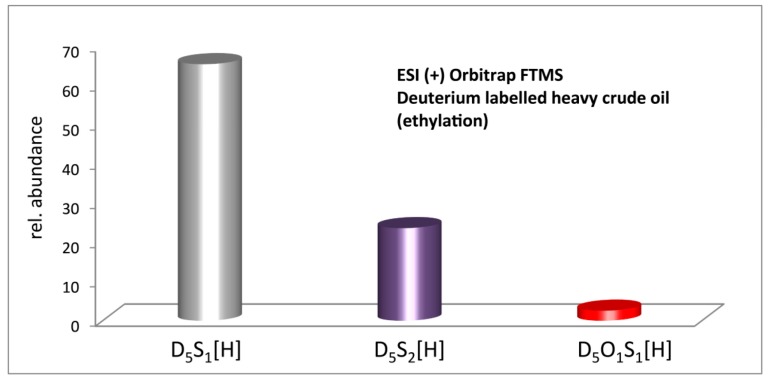
Heteroatom class distribution of heavy crude oil after deuterium labelled ethylation.

Additional ESI(+) FT-ICR MS analyses were performed on the deuterated ethylated heavy crude oil, as shown in Figure 6. Here, the results of the ethylation reaction are consistent with those from the methylation reaction. The nonpolar sulfur-containing compounds (S_1_, S_2_ and S_1_O_1_ species) can be selectively detected by ESI(+) and differentiated by the C_2_D_5_-group from the original crude oil. In comparison with the methylation procedure, a small difference in S_1_O_1_ class (2.5%) distribution appears which could result from different reactivities of the alkylating reagents. Still in both cases the S_1_ class is the dominant class in both alkylation reactions, followed by the S_2_ class.

**Figure 7:**
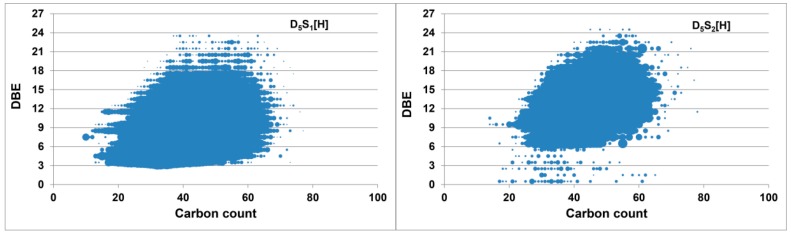
DBE *versus* carbon number plots for the D_5_S_1_ and D_5_S_2_ classes from the deuterated ethylated heavy crude oil.

When comparing the methylation reactions, the deuterated S_1_ class in the ethylation extends a slightly narrower DBE range of 4–24, and the S_2_ class has a slightly wider DBE range from 1 to 25 as demonstrated in Figure 7. The slight difference of the DBE range for S_1_ and S_2_ classes in methylation and ethylation might lie in the slight imparity of heteroatom class distribution of the two cases.

When considering the results from this study, the most interesting point is that sulfur containing compounds in a very complex crude oil mixture can be selectively analyzed by ESI-MS after derivatization. While in theory, the pyridine nitrogen is a better nucleophile than the thiophene sulfur to react in an S_N_2 reaction with alkylating reagents in the polar aprotic solvent (DCE), things change when Ag^+^ ions are involved in the reaction with crude oil. Here, the coordination chemistry of the silver ions plays an important role to kinetically favor the sulfur atoms and form weak silver-ligand bonds. When we then add the alkyl iodide to the above system, the relatively weak metal-ligand bond will be broken and a much stronger S-C covalent bond will be formed accompanied by the strong thermodynamic drive of releasing solid silver iodide (AgI) from the solution. The generated AgI could also further react with nitrogen compounds, to form insoluble coordination compounds of larger size that can subsequently be removed from the system [29]. An important factor for the better selectivity toward sulfur compounds in crude oil is therefore, the right amount of AgBF_4_.

## 3. Materials and Methods

### 3.1. Alkylation

DBT, DCE, AgBF_4_, anthracene (ANTH), dibenzofuran (DBF), acetic acid (AcOH) and dichloromethane (DCM) were purchased from Sigma-Aldrich (high purity, St. Louis, MO, USA). C_2_H_5_I, acridine (ACR), CD_3_I and C_2_D_5_I were purchased from Sigma-Aldrich (high purity, Steinheim, Germany). A heavy crude oil of North American origin was used.

10 mg of DBT were dissolved in 1 mL anhydrous DCE. During mixing, a solution of 2 molar eq. AgBF_4_ (21.13 mg) in 0.5 mL DCE was added. After 2 min, 8 molar eqivalents (28 μL) of C_2_H_5_I was then added and yellow silver iodide precipitated immediately. After 4 h, the solid was removed by centrifugation and washed with 0.5 mL of DCE. A final concentration of 500 μg/mL of the ethylated DBT was obtained through dilution with DCM; the procedure above was successively applied to a mixture of standard compounds: 6.5 mg of ANTH, 2.75 mg of DBT, 0.25 mg of ACR, 0.5 mg of DBF. The same procedure was further employed to 10 mg of the heavy crude oil by replacing C_2_H_5_I with CD_3_I and C_2_D_5_I. For a comparison, a heavy crude oil sample with a final concentration of 500 μg/mL in DCE containing 0.2% AcOH was used for ESI analysis.

### 3.2. Nuclear Magnetic Resonance Spectroscopy

^1^H-NMR spectra were recorded on a Bruker Advance 300 NMR spectrometer. The chemical shifts and the coupling constants were obtained through analysis of the spectra using Bruker TopSpin NMR-Software version 2.1 (Karlsruhe, Germany). The chemical shifts of ^1^H-NMR spectra is reported as in units of parts per million (ppm). The ^1^H-NMR spectrum is referenced through the solvent lock (^2^H) signal according to IUPAC (International Union of Pure and Applied Chemistry) recommended secondary referencing method and the manufacturer’s protocols based on impurity of CHCl_3_ in CDCl_3_. Multiplicities were given as: s (singlet); br s (broad singlet); d (doublet); t (triplet); q (quartet); m (multiplets), *etc.* The number of proton (*n*) for a given resonance is indicated by nH. The ethylated DBT was evaporated to dryness under reduced pressure to remove DCE and the excess of C_2_H_5_I. The dry ethylated DBT was then dissolved in 0.5 mL of CDCl_3_ was used for for ^1^H-NMR analysis.

### 3.3. ESI Orbitrap FTMS Analysis

Mass analyses were performed on a hybrid mass spectrometer combining the dual linear ion trap with a novel high field Orbitrap mass analyzer (LTQ Orbitrap Elite FTMS, Thermo Scientific, Bremen, Germany) [9]. Up to 500 spectra were collected in positive mode using the ESI source (Thermo Fisher, Bremen, Germany). A standard data acquisition and instrument control system was employed (Thermo Scientific, Bremen, Germany). Acquisition mass range was 150 < *m*/*z* < 1200 and the target value (AGC value) was set between 1E5 and 1E6; typical ESI conditions were as follows: flow rate 5 μL/min; spray voltage 3.0 kV; a sheath gas flow of 5 (arbitrary unit), an auxiliary gas flow of 2 (arbitrary unit). MS data were recorded with a resolving power of 480,000 at *m/z* 400 using a 1.5 s transient and up to 900,000 at *m*/*z* 400 using transient signals of 3 s for comparison. These parameters allow separating all important mass splits throughout the detected scan range. The instrument was calibrated with the Thermo Scientific Pierce LTQ Velos ESI positive ion calibration solution. In addition, external calibration was performed using a mixture of the Agilent electrospray calibration solution with masses at 300.04812, 622.02896, 922.00980, thus the whole mass range was covered in the samples. The mass accuracy below 1 ppm and the resolving power up to 480,000 at *m*/*z* 400 allows the analysis of the crude oil by the high-field Orbitrap.

### 3.4. ESI FT-ICR MS Analysis

Corresponding mass analysis was performed on a 7 T linear quadrupole ion-trap (LTQ) FT-ICR MS (Thermo Fisher, Bremen, Germany). The same ESI source was employed. Mass acquisition, ESI condition, AGC control and mass calibration follow the same setting implemented on ESI Orbitrap FTMS.

### 3.5. Data Analysis

The obtained mass data were imported into Composer software V1.06 (Sierra Analytics, Modesto, CA, USA). The following chemical constraints were applied: number of H 1000, 0 < D < 3 for reaction with CD_3_I and 0 < D < 5 for reaction with C_2_D_5_I, 0 < C < 100, 0 < S < 3, 0 < O < 3, 0 < N < 3, and 0 < double bond equivalent (DBE) < 40, with a maximum mass error of 1.5 ppm. The calculated molecular formulas were sorted into their heteroatom class (N_n_O_o_S_s_) according to their denoted Kendrick mass defects, double bond equivalence (DBE = number of rings plus double bonds involving carbon) distribution, and carbon number distribution [30].

## 4. Conclusions

Ultrahigh-resolution FT-ICR MS is an undoubtedly powerful analytical method for the analysis of incredibly complex petroleum samples. Nowadays, the high-field Orbitrap FTMS with a resolving power of up to 900,000 at *m*/*z* 400 is becoming an attractive alternative to FT-ICR MS for petroleum analysis as shown in this work and others [9,10]. Different chemical methods, such as protonation and alkylation, have certain selectivity toward some classes of compounds in crude oil, and although MS has an unmatched advantage for the analysis of petroleum-type samples, it is still not able to fully characterize such tremendously complex mixtures. The combination of various selective methods [31], such as chemical derivatization, chromatographic methods, *etc.*, might make a comprehensive characterization of a crude oil possible. Herein, by combining the coordination chemistry and organic chemistry together with the ultrahigh resolution mass spectrometry (FT-ICR MS and high-field Orbitrap FTMS) coupled with positive ESI, we demonstrate a highly selective method toward sulfur-containing compounds analysis in a whole heavy crude oil without any fractionation and chromatographic separation. Deuterium labeling is utilized to introduce specific information of isotopically labeled atoms which are not present at high abundance in crude oil and allow unambiguous differentiation of the nonpolar sulfur species formed from CD_3_ or C_2_D_5_ from the original crude oil in positive ESI. Using the labeling procedure also allows to fully assigning all reacted components within the very complex crude oil mixture, thus allowing full characterization of the reaction.
